# Comparison of three embryo culture media: A randomized clinical trial with sibling oocytes

**DOI:** 10.5935/1518-0557.20260025

**Published:** 2026

**Authors:** Bruna Campos Galgaro, Lisiane Knob de Souza, Luíza da Silva Rodrigues, João Sabino Lahorgue da Cunha-Filho

**Affiliations:** 1 Insemine Center of Human Reproduction, Porto Alegre, Brazil; 2 Department of Gynecology and Obstetrics, Faculty of Medicine, Universidade Federal do Rio Grande do Sul, Ramiro Barcelos 2400, 90035-003 Porto Alegre, Brazil

**Keywords:** culture media, fertilization, embryo quality, continuous media, sequential media

## Abstract

**Objective:**

To evaluate the influence of three culture media on the good quality cleavage embryo and fertilization rates.

**Methods:**

A three-arm, prospective, randomized, controlled clinical trial included patients undergoing IVF treatments at a single center from May to August 2023. Sibling oocytes were randomized in three culture media (A, B and C). Fertilization and day three good quality embryo rates were evaluated.

**Results:**

Three hundred sixty-four retrieved oocytes from forty patients were randomized right after ovarian aspiration. From those, 309 metaphase II oocytes (mean per patient 7.72±2.87) were fertilized and cultured. Overall fertilization rate was 81.6%, with no statistical difference among media: 85.8% (73-100) *vs*. 77.2% (63-100) *vs*. 82.4% (67-100) for A, B and C, respectively (*p*=0.290). Although the percentage of D3 good quality embryos in each media was not statistically significant [A: 52.3% (0-76), B: 35.9% (0-67), C: 44.8% (0-100) (*p*=0.137)], the number of patients with at least one good quality embryo on D3 was statistically different [28 (70.0%) *vs*. 18 (45.0%) for A and B, respectively (p=0.020) OR 1.56; 95% CI (1.05-2.32, *p*=0.020)]. In a multivariable analysis, culture media and age have significantly affected D3 embryo quality OR 1.44; 95% CI (1.22-2.35, p=0.016).

**Conclusions:**

Although no difference was observed in fertilization and cleavage-stage embryo quality rate among brands, one culture medium had a significantly lower number of patients with good-quality cleavage embryos. Further trials with pregnancy outcomes are warranted to confirm clinical implications of these findings.

## INTRODUCTION

**Figure:**
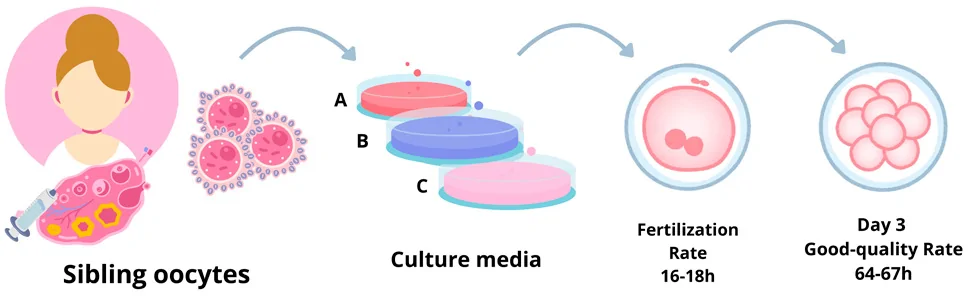

Optimal embryo culture is critical for achieving improved *in vitro* fertilization (IVF) outcomes. Nonetheless, multiple factors can negatively impact embryonic development: Plastic labware, osmolality, pH, gas concentration, oil overlay, temperature, and the culture media. These laboratory-related variables need validation and continuous quality control to ensure positive clinical outcomes (Bossi *et al*., 2023; Hardarson *et al*., 2015; Hong *et al*., 2014; Mestres *et al*., 2021).

Currently, there are numerous options of culture media formulations for in vitro embryonic development. Their composition is elaborated according to different culture strategies: sequential and continuous. The sequential culture method is designed to replicate the conditions of the fallopian tube and uterus, delivering the necessary nutrients to the embryo at each stage of development. For this reason, sequential media contain a higher concentration of lactate and pyruvate for the cleavage stage. In contrast, the glucose concentration is higher in the second medium for culture up to blastocyst (Gardner *et al*., 1996; Sciorio & Rinaudo, 2023).

Conversely, the continuous culture strategy enables the embryo to utilize available energy substrates in accordance with its metabolic requirements at each stage of development. One of the main advantages of this system is the reduction of dish manipulation and embryo stress (Máté & Török, 2020; Reed *et al*., 2009). Thus, the composition of carbohydrate sources and amino acids concentration in the medium varies according to the manufacturer and the culture policy (Morbeck *et al*., 2014; Tarahomi *et al*., 2019).

Some recent studies demonstrate that culture media have a direct impact on embryonic and placental quality and epigenetics (Kleijkers *et al*., 2015; Mulder *et al*., 2020; Sunde *et al*., 2016; Tao *et al*., 2022), and even on the weight of newborns (Dumoulin *et al*., 2010; Kleijkers *et al*., 2016; Sacha *et al*., 2022; Zandstra *et al*., 2015). Nonetheless, the randomized clinical trials conducted to date have been unable to determine the optimal culture medium in terms of embryo development, aneuploidy rates, or clinical outcomes. (Mantikou *et al*., 2013; Sfontouris *et al*., 2016; Sfontouris *et al*., 2017a).

Elucidating the role of the culture medium is crucial for promoting optimal embryonic development and supporting a successful pregnancy. Therefore, the present study aimed to assess the effects of three commercially available culture media-one sequential and two continuous-on fertilization and good-quality cleavage-stage embryo rates. For this purpose, a randomized clinical trial was conducted using sibling oocytes from patients undergoing IVF treatment.

## MATERIAL AND METHODS

### Overall study design

**Figure 1:**
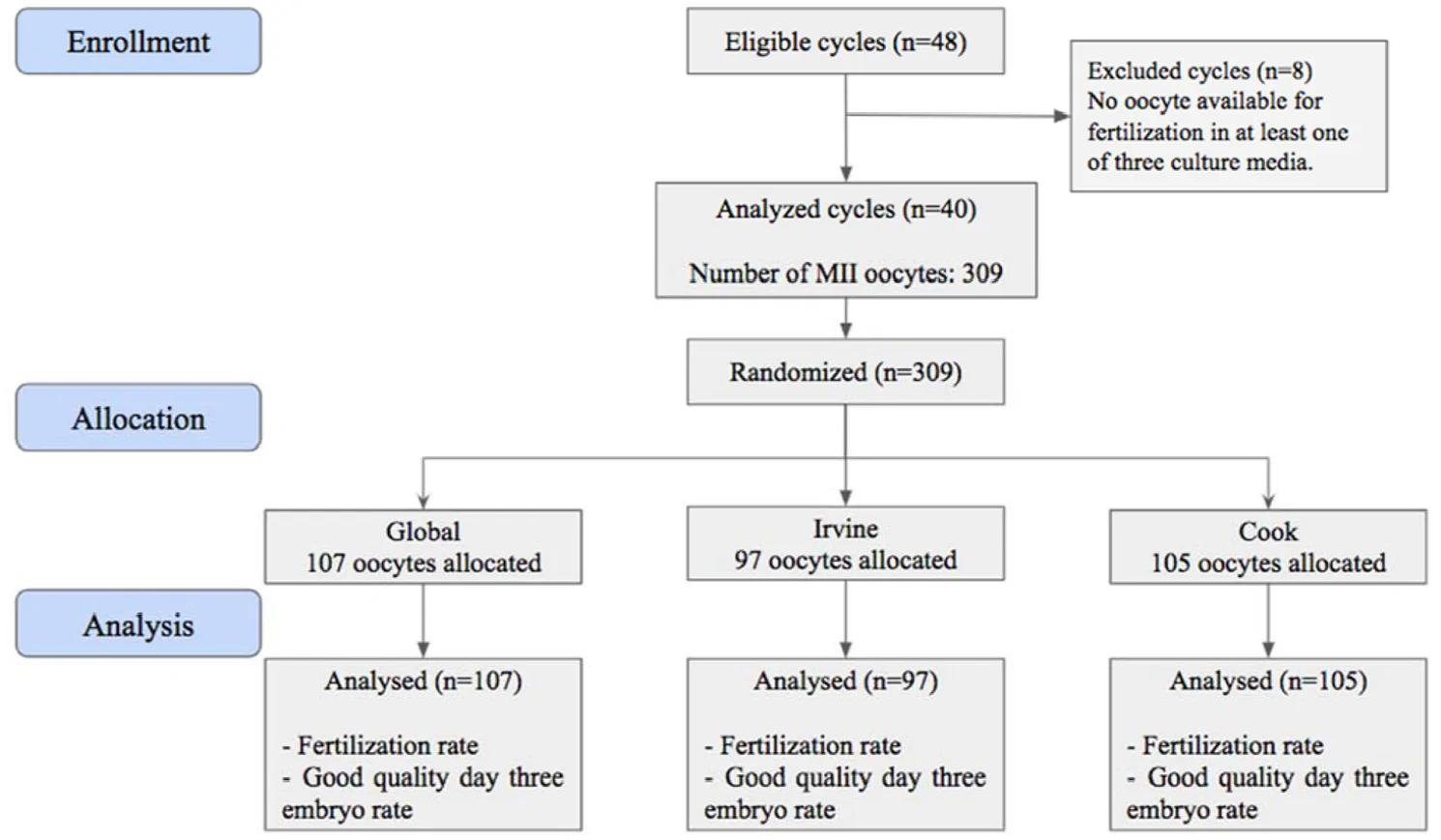
Study population, randomization, and analysis

A three-arm, prospective, randomized, controlled clinical trial included patients undergoing IVF treatments at a single center, from May to August 2023. The Research Ethics Committee of Hospital de Clínicas de Porto Alegre approved the study on 08/24/2022, as the National Health Council recommended, and registered at ClinicalTrials.gov (NCT05676515) on 12/22/2022. Trial registration was completed after initiation due to administrative delay. The first and last patients were enrolled on May 7, 2023, and August 23, 2023. As shown in Figure 1, we prospectively randomized sibling oocytes right after ovarian aspiration in three different culture media (Global Total LP, «Global» from Life Global - medium A; Continuous Single Culture Media Complete, «Irvine» from Irvine Scientific - medium B; and Sidney IVF Cleavage Media, «Cook» from Cook Medical - medium C) and compared fertilization and day three (D3) embryo quality rates.

The study’s primary outcome was the good-quality embryo rate at D3; the secondary was the fertilization rate. We decided not to evaluate the blastocyst rate because some patients’ embryos are transferred or frozen at D3, and not all undergo extended culture. Our practice aligns with most European clinics, as 55.9% of fresh transfers are reported at the cleavage stage (European IVF-Monitoring Consortium (EIM) for the European Society of Human Reproduction and Embryology (ESHRE) *et al*., 2021).

### Recruitment and sample size

Couples undergoing IVF treatment for idiopathic, male or female causes were eligible. The included criteria were patients with at least three oocytes retrieved in a single ovarian aspiration and with a signed informed consent form. The exclusion criteria was less than three oocytes available to perform fertilization.

Considering a difference of 30% between groups in the number of good quality embryos (A + B) (ESHRE Special Interest Group of Embryology and Alpha Scientists in Reproductive Medicine, 2017) with the power of 80% and p alpha 5 %, the calculated n was 95 oocytes for each culture medium, totaling 285 oocytes retrieved and fertilized by intracytoplasmic sperm injection (ICSI). Due to the average number of oocytes retrieved per patient at this center being six, we calculated that 48 patients would be needed to reach the final number of 285 oocytes.

### Stimulation Protocol

All patients underwent controlled ovarian stimulation. A range dose of 150-300 IU of HMG (Menotropina, Menopur, Ferring) was administered in combination with a gonadotropin release hormone antagonist (Ganirelix, 25 mcg/day, Orgalutran, Schering-Plough) started on day six of the ovulation cycle. When at least three follicles measured 17mm in diameter, a 5,000 IU dose of HCG (Choriomon, Biopharma) was administered. Oocyte pick-up was performed 36 hours after HCG triggering by ultrasound-guided ovarian aspiration.

### Media preparation

All media dishes were prepared on the day before the ovarian aspiration. For fertilization dishes, where oocytes were placed after pick-up until the ICSI procedure, dishes were composed of 100µL drops of respective medium. For embryo culture, media dishes were prepared with three 100µL drops for washing the oocytes after ICSI and individual 50µL drops for each embryo. All plates were covered with 10mL of paraffin oil (FertiPro) and incubated for equilibration for approximately 18 hours at 37ºC with 6% CO_2_. Media pH were measured with I-stat CG4+ cartridges (Abbott) for each batch.

### Randomization

Oocytes retrieved from the same patient (sibling oocytes) were randomized (random numbers from sealedenvelope.com) in three culture media in a ratio of 1:1:1 right after ovarian aspiration by the embryologist responsible for the procedure. Once randomized, there was no manipulation or reallocation between groups. They were fertilized from three to six hours post oocyte pick-up, and the embryos formed were cultured until the day of transfer or embryo freezing. The fertilization rate was evaluated 16 to 18 hours after fertilization, and the embryonic quality was assessed between 64 and 67 hours. Both clinicians and couples were blinded during this study.

### Analyzed variables

Fertilization rate: The presence of two pronuclei and two polar bodies (2PN/2PBs) indicated fertilization. The fertilization rate was evaluated by the percentage of 2PN/2PBs zygotes observed in the check between 16 and 18 hours after fertilization by ICSI.

Embryo quality: At D3 (64-67 hours after fertilization), embryos were classified as A, B, C, or D according to parameters of cell number, symmetry, and fragmentation. A and B embryos (7-9 cell, symmetric, and fragmentation ≤20%) were considered good quality. Embryo quality was defined as the primary outcome of this study because it is a parameter directly related to the prognosis of IVF treatment, has a predefined value in the Vienna consensus, and is commonly described in scientific articles.

Female age, anti-mullerian hormone (AMH), and cause of infertility were obtained via the patients’ electronic medical records.

### Statistical analysis

Clinical variables with symmetric and asymmetric distribution were described as mean±standard deviation (SD) and median with 25th and 75th percentiles, respectively. Fertilization and good-quality embryo rates were expressed in percentage (mean and interquartiles 25-75). ANOVA and chi-square tests were used to compare fertilization and embryonic quality between the culture media, and a significant difference was considered if *p*<0.05. Multivariable analysis used day-3 embryo quality (at least one embryo with good quality) as the dependent variable, controlled by age, AMH, and culture media (Global, Irvine, or Cook) to describe the individual effect of those variables on embryo development. This result was presented with odds-ratio (OR) and a 95% confidente interval (CI). For tabulation and statistical analysis, the JASP software version 0.16.2 was used.

## RESULTS

In the recruitment period, 48 patients were eligible for this study. After ovarian aspiration, eight were excluded because no oocyte was available for fertilization in at least one media. Therefore, 40 ICSI cycles met all the criteria and were included (Figure 1).

Table 1 describes the clinical data of the patients. The mean age was 35.82±6.01 years, and the median AMH level was 2.24 (1.42-4.17). Seventy-five percent had primary infertility, and the leading exclusive cause was anatomic female factor (n=7).

**Table 1:** Clinical data of patients

Clinical data	Analyzed cycles (n=40)
Woman's age (years)	35.82±6.01 ^a^
AMH level (ng/mL)	2.24 (1.42-4.17) ^b^
Type of Infertility, n (%) Primary Secondary	30 (75) 10 (25)
Cause of infertility, n (%) Endometriosis Ovarian insufficiency Polycystic ovary syndrome Anatomic female factor Masculine factor Others Multiple causes	4 (10) 3 (7.5) 1 (2.5) 7 (17.5) 6 (15) 3 (7.5) 16 (40)

a Mean±SD

b Median (25^th^ and 75^th^ percentiles)

**Figure 2:**
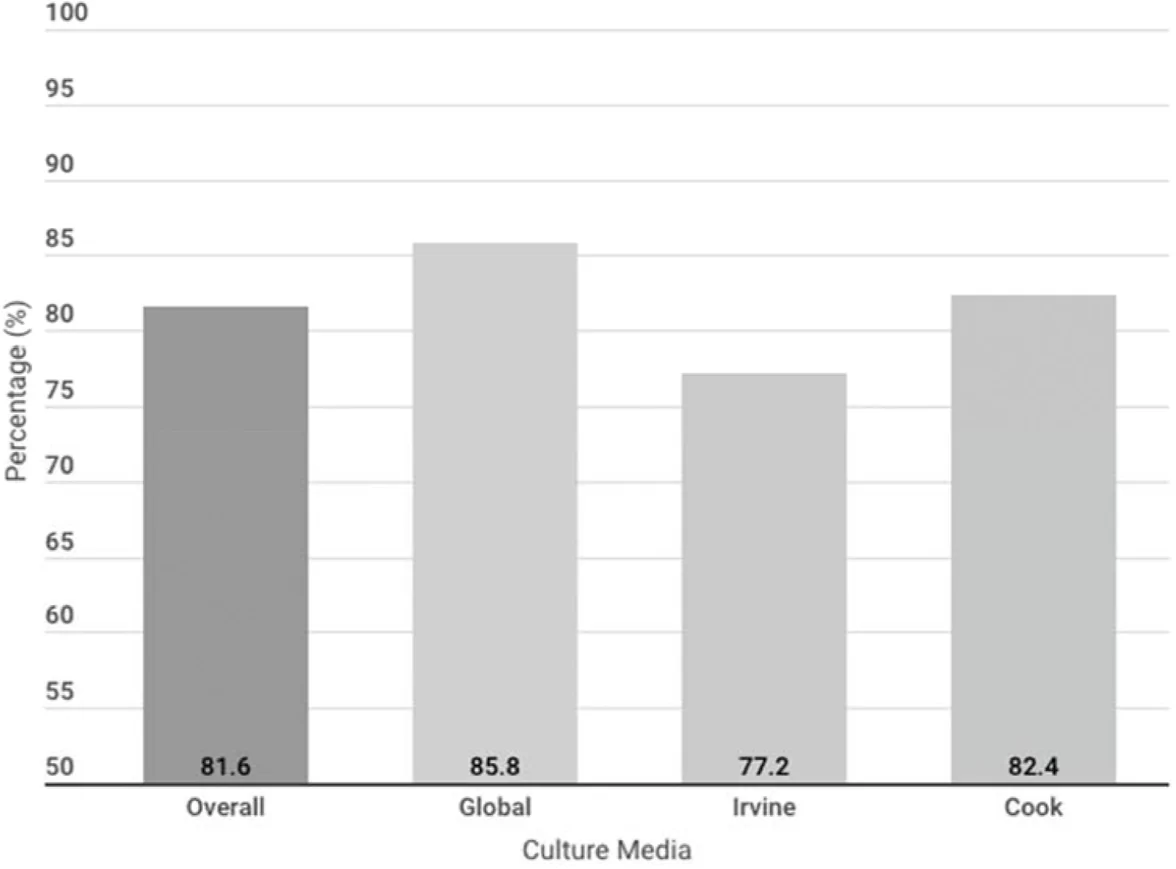
Fertilization rate

In total, 364 retrieved oocytes were randomized right after ovarian aspiration. Of these, 309 MII oocytes (mean per patient 7.72±2.87) were fertilized and cultured in three different commercial media: 107 in Global, 97 in Irvine and 105 in Cook. As shown in Figure 2, the overall fertilization rate was 81.6%. There was no statistical difference among media (mean and interquartiles 25-75): 85.8% (73-100) *vs*. 77.2% (63-100) *vs*. 82.4% (67-100) for Global, Irvine, and Cook, respectively (*p*=0.290).

There was no difference in fertilization rate among medium brands. ANOVA test (*p*=0.290). Data are shown as mean (%) for Global, Irvine, and Cook, respectively.

**Figure 3:**
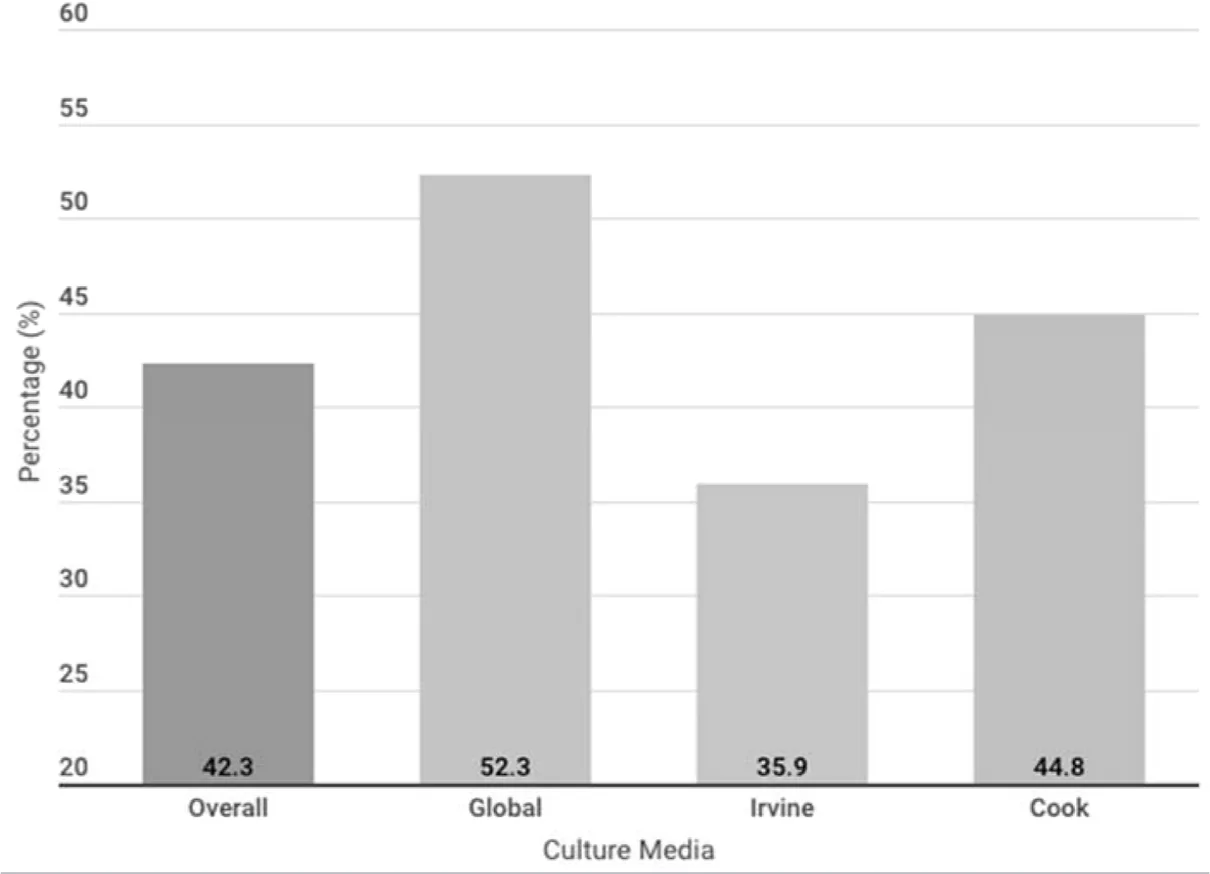
Good quality D3 embryo rate

There was no significant difference in the rate of D3 good-quality embryos (Figure 3). The percentage of A and B embryos in each media was (mean and interquartiles 25-75): Global 52.3% (0-76) *vs.* Irvine 35.9% (0-67) *vs.* Cook 44.8% (0-100) (*p*=0.137). However, the number of patients with at least one good-quality embryo on D3 was statistically different: 28 (70.0%) *vs*. 18 (45.0%) for Global and Irvine, respectively OR 1.56; 95% CI (1.05-2.32, *p*=0.020).

There was no difference in fertilization rate among medium brands. ANOVA test (*p*=0.137). Data are shown as mean (%) for Global, Irvine, and Cook, respectively.

Finally, a logistic regression analysis, considering age, AMH, and the type of media as independent variables, controlling for at least one D3 good quality embryo development as the dependent variable, showed a significant effect of age and media significantly associated with D3 embryo quality OR 1.44; 95% CI (1.22-2.35, *p*=0.016).

## DISCUSSION

The success of IVF treatment relies on a combination of biological and technical factors that demand continuous laboratory quality control. Currently, an expanding range of laboratory equipment and consumables is available, including at least fifteen commercially distributed preimplantation human embryo culture media. These media differ in characteristics such as osmolality, molecular composition, and component concentrations (Tarahomi *et al*., 2019). Since the optimal composition is still unknown, each IVF laboratory establishes its own protocol based on factors such as incubator type, cost, availability, and gas composition. Therefore, comparing different culture systems is challenging to reproduce among clinics worldwide.

Here, we investigated three commercial IVF culture media for preimplantation embryonic development, aiming to compare fertilization rates and Day 3 good-quality embryo outcomes. To date, the literature contains conflicting data due to several confounding laboratory factors that go far beyond the composition of culture media. Furthermore, biological factors related to patient characteristics may affect IVF outcomes. Therefore, we designed this experimental study using sibling oocytes to reduce a potential bias due to oocyte quality.

We evaluated fertilization and Day 3 good-quality embryo rates due to their relevance in assessing technical proficiency and overall laboratory performance (ESHRE Special Interest Group of Embryology and Alpha Scientists in Reproductive Medicine, 2017; Vaiarelli *et al*., 2023). In contrast to blastulation and usable blastocyst rates, cleavage embryo development and fertilization rates are not related to the patient’s age (Zacà *et al*., 2022), making those indicators more applicable. For instance, normal fertilization rate has been reported as a strong independent predictor of implantation (Rosen *et al*., 2010) and cumulative live birth rate (Scaravelli *et al*., 2021).

Although blastocysts have higher pregnancy outcomes per transfer compared with cleavage-stage embryos (Glujovsky *et al*., 2022), data are still controversial in terms of cumulative live birth rates (CLBR) per ovarian aspiration. No difference in CLBR between single blastocyst and cleavage transfer was observed in women with at least four embryos (Cornelisse *et al*., 2024). Whereas double cleavage-stage led to higher CLBR compared with single blastocyst transfer in patients with less than five good-quality embryos (Han *et al*., 2024). Moreover, a large multivariate analysis demonstrated that the total number of good-quality cleavage-stage embryos is the strongest predictor of clinical pregnancy following an IVF/ICSI cycle (Cai *et al*., 2011). More recently, an increased number of embryo cells on Day 3 was associated with higher clinical pregnancy and live birth rates after single blastocyst transfer (Wang *et al*., 2022). Finally, by limiting our analysis to the cleavage phase, we minimized other potential biases related to blastocyst development, such as embryonic genome activation - which occurs at the 8-cell stage (Yuan *et al*., 2023)-, and the change to a low oxygen tension incubator, which is related to a better blastulation rate (Meintjes *et al*., 2009).

The culture media included in this study were chosen because they are commonly employed in IVF clinics. Although manufacturers do not disclose the exact composition of each formulation, previous research identified two main differences between them (Morbeck *et al*., 2014). First, the strategy of the culture system leads to distinguished concentrations of the carbohydrate sources and the lactate/pyruvate ratio. Single-step media (Global and Irvine) have a higher lactate/pyruvate ratio than sequential media (Cook). Second, the composition of amino acids, which are fundamental for many cellular processes and support preimplantation embryo development (Houghton, 2012), also varies. Finally, Cook media presents a high concentration of nonessential amino acids and a low concentration of essential amino acids, whereas Global and Irvine culture media exhibit the opposite (Morbeck *et al*., 2014). In addition, each medium has its optimal pH range indicated by the manufacturer: Cook 7.30-7.35; Irvine 7.28-7.30; Global 7.29-7.30. In this work, all media used were validated after pH measurement (Cook 7.305 - 7.338; Irvine 7.294 - 7.309; Global 7.289 - 7.303).

Our results showed no differences in fertilization rates or in overall Day 3 good-quality embryo outcomes among the three formulations tested. Consistent with previous reports, the continuous media from the two commercial brands demonstrated equivalent performance regarding fertilization, embryonic development, and embryo quality (Abdala *et al*., 2021; Sfontouris *et al*., 2017b). However, we observed a significant difference when analyzing the number of patients with at least one good-quality embryo on D3. Interestingly, Irvine had fewer patients with at least one good-quality embryo, and this rate was remarkably under the competency value of 45% recommended by the Vienna consensus (ESHRE Special Interest Group of Embryology and Alpha Scientists in Reproductive Medicine, 2017). This finding could indicate that this formulation does not support the optimal culture condition for some patients.

A possible explanation may be related to the glucose concentration in the media. Glucose is the third energy source, and previous studies indicate that its presence in the early stages could be detrimental to embryo development (Coates *et al*., 1999; Conaghan *et al*., 1993). The Irvine routine uses the same media for fertilization and the whole culture, with a constant high glucose concentration. Instead, both Global and Cook brands have a unique formulation with higher glucose levels, used only to incubate oocytes and spermatozoa before/during the fertilization technique. For embryo culture, Global and cleavage stage Cook media have lower glucose concentration (Morbeck *et al*., 2014; Tarahomi *et al*., 2019). Therefore, glucose concentration may dictate embryonic fate in the first divisions, leading to poor development when elevated.

Additional studies using sibling oocytes have shown that continuous and sequential culture media yield comparable rates of usable blastocysts, as well as similar euploid and mosaic outcomes in PGT-A cycles (Abdala *et al*., 2021, 2023). In contrast, a retrospective study comparing two culture media systems reported, across all evaluated cycles, increased blastocyst formation accompanied by a higher aneuploidy rate in the single-step group (Deng *et al*., 2020). Consistently, a meta-analysis including extended culture protocols demonstrated greater blastocyst development in continuous media; however, it did not identify the optimal system with respect to clinical pregnancy, miscarriage, or ongoing pregnancy rates (Sfontouris *et al*., 2016).

Indeed, this debate has persisted for decades, and the introduction of time-lapse systems has emerged as a promising approach for embryo selection and as a source of developmental insights through kinetic analysis. A retrospective observational study compared the morphokinetics of cleavage-stage embryos cultured in single-step versus sequential media. Although all cleavage events occurred earlier in the single-step medium, clinical pregnancy outcomes were similar between the groups (van Duijn *et al*., 2022). Other recent studies have likewise reported differences in preimplantation embryo developmental kinetics according to the culture media brand, without any corresponding impact on euploidy or clinical outcomes (Ma *et al*., 2022; Urich *et al*., 2022).

This study has some limitations. The small sample size may lead to an overinterpretation of Irvine’s poorer performance, which should be assessed in larger clinical trials. Furthermore, we included patients with at least three oocytes available for fertilization, therefore, these data need further validation for poor responders. Also, we did not evaluate the whole embryo culture system. Since in our practice only some of the patient’s embryos undergo extended culture, the blastulation rate was not assessed. However, it is worth mentioning that only 44.1% of fresh transfers are at the blastocyst stage (European IVF-Monitoring Consortium (EIM) for the European Society of Human Reproduction and Embryology (ESHRE) *et al*., 2021), which sustains the importance of cleavage embryo data. Moreover, although the best outcome of clinical relevance is pregnancy rate, we did not evaluate it because of the study’s design. If we intended to compare pregnancy rates, we would not have the patients controlled by sibling oocytes.

## CONCLUSION

This randomized trial suggests that while overall fertilization and cleavage-stage embryo quality did not differ between media, one medium was associated with fewer patients achieving at least one good-quality embryo. While the optimal formulation for supporting embryo development is still uncertain, larger multicenter trials with clinical outcomes are warranted.
